# Are early first trimester weights valid proxies for preconception weight?

**DOI:** 10.1186/s12884-016-1159-6

**Published:** 2016-11-21

**Authors:** Rebecca A. Krukowski, Delia S. West, Marisha DiCarlo, Kartik Shankar, Mario A. Cleves, Marie E. Saylors, Aline Andres

**Affiliations:** 1 Department of Preventive Medicine, University of Tennessee Health Science Center, 66 N. Pauline St, Memphis, TN 38163 USA; 2 Department of Exercise Science, Arnold School of Public Health, University of South Carolina, Columbia, SC USA; 3 Arkansas Department of Health, Office of Health Communications, Little Rock, AR USA; 4 Department of Pediatrics, Arkansas Children’s Nutrition Center, University of Arkansas for Medical Sciences, Little Rock, AR USA; 5 Pediatrics Biostatistics, Arkansas Children’s Nutrition Center, University of Arkansas for Medical Sciences, College of Medicine, Little Rock, AR USA; 6 Pediatrics Biostatistics, University of Arkansas for Medical Sciences, College of Medicine, Little Rock, AR USA

**Keywords:** Obesity, Obstetrics, Preventive medicine

## Abstract

**Background:**

An accurate estimate of preconception weight is necessary for providing a gestational weight gain range based on the Institute of Medicine’s guidelines; however, an accurate and proximal preconception weight is not available for most women. We examined the validity of first trimester weights for estimating preconception body mass index category.

**Methods:**

Under identical measurement conditions, preconception weight and two first trimester weights (i.e., 4–10 and 12 weeks gestation) were obtained (*n* = 43).

**Results:**

The 4–10 week and the 12 week weight correctly classified 95 and 91% women, respectively. Mean weight changes were relatively small overall (*M* = 0.74 ± 1.99 kg at 4–10 weeks and *M* = 1.02 ± 2.46 at 12 weeks). There was a significant difference in mean weight gain by body mass index category at 4–10 weeks (−0.09 ± 1.86 kg for normal weight participants vs. 1.61 + 1.76 kg for overweight/obese participants, *p* = 0.01), but not at 12 weeks (0.53 ± 2.29 kg for normal weight participants vs. 1.54 ± 2.58 kg for overweight/obese participants).

**Conclusions:**

Assigning gestational weight gain guidelines based on an early first trimester weight resulted in 5–9% of women being misclassified depending on the gestational week the weight was obtained. Thus, most women are correctly classified based on a first trimester weight, particularly an early first trimester weight, although it is possible that modeling strategies could be developed to further improve estimates of preconception body mass index category.

**Trial registration:**

Clinicaltrials.gov # NCT01131117, registered May 25, 2010.

## Background

Obtaining an accurate and proximal preconception weight is a challenge, as many pregnancies are not planned [1] and even if the pregnancy is planned, the amount of time between deciding to conceive and actual conception is variable. The Institute of Medicine’s (IOM) body mass index (BMI)-specific gestational weight gain (GWG) recommendations [2] are based on preconception BMI, so having an accurate estimate of preconception weight is necessary for providing an appropriate GWG recommendation.

While previous research has demonstrated strong concordance between self-reported preconception weight and clinical record of preconception weight in the past year [3], more recent analyses have demonstrated particular challenges with the accuracy of self-reported preconception weight among those with a higher BMI [4]. There has only been one previous study which compared measured preconception weight with measured weight obtained at one point in the first trimester (i.e., approximately 9 weeks gestation) ([5] with data from [6]). This study found a 1.3 kg increase in weight from the measured preconception weight to the measured first trimester weight, and almost 1 in 10 of women were misclassified into a BMI category and thus may have received an inaccurate GWG recommendation based on the first trimester weight. However, no previous investigations have assessed potential differences between measured preconception weight to measured first trimester weight by BMI category. In addition, as women present for their first prenatal visit at varying times, it may be important to examine the impact of using weights obtained at various points in the first trimester in estimating preconception BMI categorization. Thus, the objective of the current study was to determine the validity of using two different measured first trimester weights (4–10 weeks and 12 weeks), by BMI category, to estimate preconception BMI category.

## Methods

Participants were part of a longitudinal cohort, the Glowing Study (clinicaltrials.gov # NCT01131117), which is examining the effects of maternal body composition on infant birth weight, growth, body composition, and risk of overweight at 2 years old. Participants were recruited from 2011 to 2014 in a small southern city. Women were eligible if they had a single previous pregnancy, had a BMI between 18.5 and 35 kg/m^2^, and were 21 years of age or older. Exclusion criteria included having preexisting medical conditions (e.g., diabetes mellitus, hypertension), taking medications known to influence fetal growth (e.g., glucocorticoids, insulin, thyroid hormones), and planning to smoke or drink alcohol during the pregnancy. A total of 287 participants met the inclusion/exclusion criteria and were enrolled in the study. Participants were enrolled in the primary study if they were planning a pregnancy or were less than 10 weeks gestation. Of those women, 51 women completed a preconception visit and of those, 43 women had measured weight at 4–10 weeks and 12 weeks; we will focus on this subsample of 43 participants for these secondary analyses.

At the preconception visit, participants were advised to remain weight stable during the first trimester, consistent with the IOM guidelines [2]. All participants received information on the IOM’s GWG guidelines [2] tailored to their BMI category at the 4–10 weeks gestation visit as well as the rationale for GWG guidelines during pregnancy (i.e., maternal and child health). Research staff also introduced and explained a GWG graph (tailored to BMI category) that would be used to track the participant’s GWG throughout her pregnancy. During pregnancy, all participants received six behavioral intervention sessions (i.e., at 4–10, 12, 18, 24, 30, and 36 weeks gestation) designed to promote healthy GWG, with intensified intervention offered in the presence of excessive GWG. The intervention has been described in detail elsewhere [7].

Weight was measured in a hospital gown with no shoes to the nearest 0.1 kg using a calibrated tarred standing digital scale at all study visits under fasted conditions. Height was measured to the nearest 0.1 cm using a wall-mounted stadiometer at preconception only. All measures were obtained in duplicate, with a third assessment if there is discrepancy between the first two. BMI was calculated from these measures [weight(kg)/height(m)^2^], and women were classified as normal weight (*n* = 22) or overweight/obese (*n* = 21) at the preconception visit [8]. Of those participants in the overweight/obese category at the preconception visit, 17 participants were overweight and 4 were obese. Informed consent was obtained from participants, and all study procedures were approved by the Institutional Review Board of the University of Arkansas for Medical Sciences.

Descriptive statistics were calculated to describe weight change over each interval, time interval to conception, and the proportion of women correctly classified using the first trimester weights. Cohen’s kappa (κ) statistic [9] was used to assess the agreement between preconception BMI classification (i.e., normal, obese, overweight) and BMI classification at 4–10 weeks and 12 weeks of pregnancy. Bland-Altman plots [10] were used to examine the agreement between preconception BMI and the later BMIs. Both mean bias and 95% limits of agreement were computed. Because a non-significant linear trend between the difference of paired BMI values and their average was observed, Bland-Altman’s limits of agreement were not adjusted for trend. Additionally, agreement between preconception BMI and the later BMI measurements was evaluated by computing and testing Lin’s concordance correlation coefficient [11]. Lin’s concordance correlation coefficient (CCC) provides an estimate of the degree to which repeated measurements deviate from the 45° line of perfect concordance. The concordance correlation coefficient combines measures of both precision and accuracy. Weight change from preconception was compared to first trimester weights by BMI category and based on whether BMI category changed using Wilcoxon’s rank-sum (Mann–Whitney) tests. Statistical analysis was performed using Stata 14.0 statistical package (Stata Corporation, College Station, TX, USA).

## Results

The sociodemographic characteristics of the subsample of participants (*n* = 43) who had a preconception weight and measured weight at 4–10 and 12 weeks gestation was examined in relation to the primary study sample of participants (*n* = 244) who met the inclusion/exclusion criteria and enrolled in the study, but did not have a preconception weight, or a measured weight at 4–10 or 12 weeks gestation or had a miscarriage between the preconception weight and the measured weight at 4–10 weeks (Table 1). A significantly greater proportion of those included in the subsample were Caucasian and married compared to those excluded from the subsample due to missing weights at any one of the three critical measurement points. There were no significant differences between the samples in BMI categorization, age, ethnicity, or education level (*p* > 0.05).

**Table 1:** Comparison of Socio-demographic Characteristics Between the Subsample of Included Participants and Those Excluded From These Analyses

	Excluded	Included	*P*-value
	(*N* = 244)	(*N* = 43)	
Body Mass Index Category			0.16
Normal %(*N*)	43% (104)	51% (22)	
Overweight %(*N*)	36% (87)	40% (17)	
Obese %(*N*)	22% (53)	9% (4)	
Age M ± SD	31.58 ± 4.18	32.44 + 3.20	0.13
Race			0.03
Caucasian %(*N*)	86% (209)	95% (41)	
African American %(*N*)	11% (27)	0% (0)	
Other %(*N*)	3% (8)	5% (2)	
Ethnicity			0.67
Hispanic %(*N*)	4% (9)	5% (2)	
Non-Hispanic %(*N*)	96% (235)	95% (41)	
Marital Status			0.04
Missing Data %(*N*)	1% (2)	0% (0)	
Married, Biological Parent %(*N*)	86% (211)	100% (43)	
Cohabitating, Biological Parent %(*N*)	8% (20)	0% (0)	
Divorced, Single or Cohabitating, Non-biological Parent %(*N*)	5% (11)	0% (0)	
Education			0.86
Missing Data %(*N*)	1% (2)	0% (0)	
High School or GED %(*N*)	7% (16)	7% (3)	
Partial College or Graduate %(*N*)	61% (148)	56% (24)	
Graduate Training or Degree %(*N*)	28% (68)	33% (14)	
Specialized Training %(*N*)	4% (10)	5% (2)	

Participants included in the subsample analyses were 96% Caucasian and 5% Hispanic with a mean ± standard deviation age = 32.44 ± 3.20 years. The 4–10 and 12 week weights were obtained, on average, at 7.1 ± 1.5 weeks and 12.0 ± 0.8 weeks, respectively, based on self-reported date of last menstrual period. The average interval from the preconception visit to the 4–10 and 12 week visits was 120.6 ± 82.8 days and 154.7 ± 80.0 days, respectively.

**Fig. 1:**
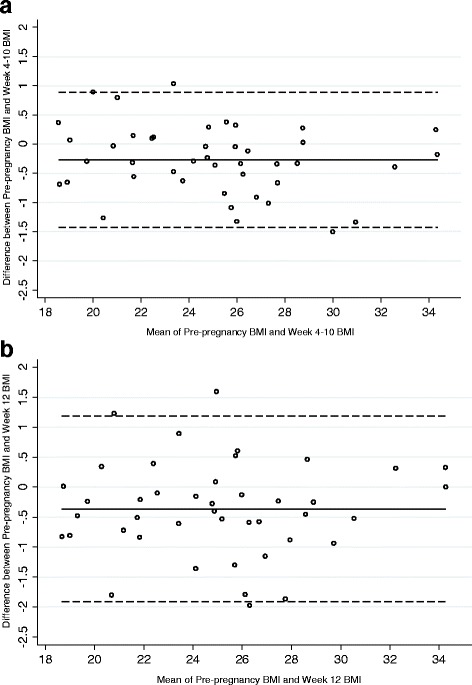
Bland Altman Plots Assessing Agreement Between Pre-Conception Body Mass Index and Body Mass Index at 4–10 Weeks Gestation (Panel **a**) and Body Mass Index at 12 Weeks Gestation (Panel **b**)

Weight change at 4–10 weeks and at 12 weeks of pregnancy overall and by BMI category are presented in Table 2. There was a significant difference in weight change by BMI category at 4–10 weeks but not at 12 weeks (Table 2). Cohen’s kappa statistics showed a high agreement between the preconception BMI classification and the BMI classification at 4–10 weeks (κ =0.92, *p* < 0.001). Very high agreement was found between the preconception BMI category and BMI category at 4–10 weeks (CCC = 0.99, 95% CI: (0.98, 0.99), *p* <0.001). The Bland-Altman’s analysis showed good agreement between preconception BMI and BMI at 4–10 weeks with minimal mean bias (−0.27) and one observation above and one observation below the 95% limits of agreement (Fig. 1, panel a). The two women (1 normal weight and 1 overweight participant at the preconception visit) who changed BMI category at 4–10 weeks both experienced a weight gain.

**Table 2:** Changes in Weight and Body Mass Index Category from Pre-Conception to Two First Trimester Timepoints

	All (*n* = 43)	Normal Weight (*n* = 22)	Overweight/Obese (*n* = 21)	*p*-value
Pre-Conception to 4–10 Week Visit Interval				
Mean (SD) Weight Gain, kg	0.74 (1.95)	−0.09 (1.67)	1.61 (2.21)	0.01
Range of Weight Change, kg	−5.05 to 4.85	−5.05 to 3.80	−0.90 to 4.85	
Women Remaining in Their Preconception Body Mass Index Category, *n* (%)	41 (95%)	21 (95%)	20 (95%)	
Pre-Conception to 12 Week Visit Interval				
Mean (SD) Weight Gain, kg	1.02 (2.46)	0.53 (2.29)	1.54 (2.58)	0.26
Range of Weight Change, kg	−5.40 to 5.50	−5.40 to 5.40	−3.80 to 5.50	
Women Remaining in Their Preconception Body Mass Index Category, *n* (%)	39 (91%)	20 (91%)	19 (90%)

Cohen’s kappa statistics showed a high agreement between the preconception BMI classification and the BMI classification at 12 weeks of pregnancy (κ =0.84, *p* < 0.001). Very high agreement was found between preconception BMI category and BMI category at 12 weeks (CCC = 0.98, 95% CI: (0.96, 0.99), *p* < 0.001). The Bland-Altman’s analysis also showed good agreement between preconception BMI and BMI at 12 weeks with minimal mean bias (−0.37) and 2 observations above and one below the 95% limits of agreement (Fig. 1, panel b). The majority of women (3 of 4) who changed BMI category at 12 weeks experienced a weight gain. Two women who changed BMI category at 12 weeks were classified as normal weight and two were classified as overweight at their preconception visit.

## Discussion

Approximately 19 out of every 20 women would have been assigned an accurate GWG goal using the weight obtained at 4–10 weeks gestation. Accuracy was slightly reduced at 12 weeks gestation, with 91% correctly classified at this measurement point, indicating the importance of obtaining a measured weight early in the first trimester in order to reduce misclassification. We also found a significant difference in weight change by BMI category at 4–10 weeks, such that normal weight participants remained largely weight stable but the overweight/obese participants gained more than a kilogram on average. However, we did not find a significant difference in weight change by BMI category at 12 weeks. While these results indicate relatively low rates of misclassification, any misclassification leads to women receiving incorrect GWG recommendations, which is a significant concern given the pregnancy and delivery complications [12–17] as well as short-term and long-term offspring health consequences [18–20] associated with excessive GWG.

These results are largely consistent with the single previous study of which we are aware, which compares preconception weight with weight obtained at approximately 9 weeks gestation ([5] with data from [6]) where a slightly larger weight change (+1.3 kg) was observed and a slightly larger proportion of women misclassified. The current report advances the field over this earlier report because we examined the impact of using two different time points for estimating preconception weight and assessed potential differences by BMI category. Our sample had a larger sample of overweight and obese participants (*n* = 21) as compared to the 8 overweight/obese participants in the previous study, which allowed us to examine these differences by BMI category. While we found a significant difference in weight change by BMI category at 4–10 weeks, it will be important to further examine differences in weight change by BMI category in a larger sample. In addition to the significant (i.e., at 4–10 weeks) or nonsignificant differences (i.e., 12 weeks) by BMI category, it is important to note the magnitude of mean weight change as well the range in weight change between the measurement points, particularly given the small sample size, which may be more meaningful than statistical significance.

The study is limited by the small subsample, the predominately Caucasian sample, and restriction of the sample to women with a BMI <35 and women carrying a second child. In addition, since most pregnancies are not planned [1], a preconception weight was only available for approximately 20% of the overall sample. A significantly greater proportion of participants included in the subsample of participants in these secondary analyses (based on the availability of preconception, 4–10 week and 12 week gestation weight) were Caucasian and married compared to those excluded from the sample. Thus, the generalization of these results to a more diverse population remains to be established. In particular, it is possible that these findings would differ among nulliparous women, as Fontaine et al.[21] found that parity was significantly associated with mean first trimester weight gains among overweight women, but not normal weight or obese women, such that multiparous women gained more than nulliparous women. In addition, all of the women in this sample were provided with the advice to remain weight stable during the first trimester, consistent with the IOM guidelines; this advice may not be widely known, so it is possible that there may be greater weight gain in the first trimester among individuals receiving “usual care” who may not receive this advice. These limitations are offset, however, by a rigorous protocol, which included with fasted measured weights.

## Conclusions

These data, consistent with previous data [5], indicate that estimating preconception BMI category using an early first trimester measured weight (approximately 7 weeks gestation) is reasonably accurate, regardless of BMI category. The accuracy of classification was slightly lower later in the first trimester (at approximately 12 weeks gestation), pointing to the importance of obtaining a measured weight early in the first trimester. However, given the large range in weight change between preconception and both early first trimester weights, it may be appropriate to consider developing and validating a mathematical model to estimate preconception weight [4, 5], in order to improve preconception weight estimate accuracy, and these results indicate that BMI category at preconception may be important variable to examine for this model. In future research, it will be important to examine the predictors of misclassification in larger samples.

## Abbreviations

BMI: Body mass index
CCC: Concordance correlation coefficient
GWG: Gestational weight gain
IOM: Institute of Medicine
